# Current Challenges of Mitochondrial Potassium Channel Research

**DOI:** 10.3389/fphys.2022.907015

**Published:** 2022-05-31

**Authors:** Bogusz Kulawiak, Adam Szewczyk

**Affiliations:** Laboratory of Intracellular Ion Channels, Nencki Institute of Experimental Biology, Polish Academy of Sciences, Warsaw, Poland

**Keywords:** mitochondria, potassium, mitochondrial potassium channels, channel inhibitors, potassium channel openers, cytoprotection, protein import

## Abstract

In this paper, the current challenges of mitochondrial potassium channels research were critically reviewed. Even though recent progress in understanding K^+^ traffic in mitochondria has been substantial, some basic issues of this process remain unresolved. Here, we focused on the critical discussion of the molecular identity of various mitochondrial potassium channels. This point helps to clarify why there are different potassium channels in specific mitochondria. We also described interactions of mitochondrial potassium channel subunits with other mitochondrial proteins. Posttranslational modifications of mitochondrial potassium channels and their import are essential but unexplored research areas. Additionally, problems with the pharmacological targeting of mitochondrial potassium channel were illustrated. Finally, the limitation of the techniques used to measure mitochondrial potassium channels was explained. We believe that recognizing these problems may be interesting for readers but will also help to progress the field of mitochondrial potassium channels.

## Introduction

The first mitochondrial potassium-selective channel, namely, the ATP-sensitive potassium (mitoK_ATP_) channel, was discovered over 30 years ago (Inoue et al., 1991). Application of patch-clamp technique also revealed the sensitivity of the mitoK_ATP_ channel to antidiabetic the sulfonylurea—glibenclamide. These properties suggest that in the inner mitochondrial membrane, there are potassium channels similar to the ATP-sensitive potassium (K_ATP_) channels present in the plasma membrane of various cells (Inoue et al., 1991; Kravenska et al., 2021). Initial observations were focused on the functional characterization of the mitoK_ATP_ channel. For many years, the task of this channel was not in line with Peter Mitchell’s chemiosmotic theory. This is because ion channels that dissipate the electric potential of mitochondria lower the synthesis of ATP by mitochondria. Later, the mitochondrial potassium channels were joined with cytoprotection phenomena and positioned as molecular representation of the potassium uniport, and this helped to reveal that these proteins are central part of mitochondrial ion transport complexes. This was the beginning of the story on mitochondrial potassium channels. Additionally, it is important to remember that influx of K^+^ into mitochondria is coupled with efflux of potassium via K^+^/H^+^ antiport forming so called K^+^ cycle (Garlid and Paucek 2003; Pereira and Kowaltowski 2021).

Later, the identification of several other mitochondrial potassium channels such as mitochondrial large-conductance calcium-activated potassium (mitoBK_Ca_) channels, mitochondrial intermediate-conductance calcium-activated potassium (mitoIK_Ca_) channels, mitochondrial small-conductance calcium-activated potassium (mitoSK_Ca_) channels, mitochondrial sodium-activated potassium (mitoSlo2) channels, mitochondrial voltage-regulated potassium (mitoKv) channels and mitochondrial two pore domain potassium (mitoTASK) channels was reported (Laskowski et al., 2016; Krabbendam et al., 2018; Kravenska et al., 2021). The properties, pharmacology and function of these proteins were discussed in a set of review publications summarized in Table 1.

**TABLE 1:** Review papers on mitochondrial potassium channels published since 2012 till 2022.

Topic	Details	Reviews
General information on mitochondrial potassium channels	1. Summary information on mitochondrial potassium channels	Checchetto et al. (2016), Laskowski et al. (2016), Checchetto et al. (2018), Jarmuszkiewicz and Szewczyk (2019)
	2. Mitochondrial ATP-sensitive potassium channel	Wojtovich et al. (2013), Kravenska et al. (2021)
	3. Mitochondrial calcium-activated potassium channels	Singh et al. (2012), Balderas et al. (2015), Krabbendam et al. (2018), Gonzalez-Sanabria et al. (2021)
	4. Mitochondrial voltage gated potassium channels	Bachmann et al. (2020)
	5. Non-mammalian mitochondrial potassium channels	Pastore et al. (2013), Trono et al. (2015)
Function of mitochondrial potassium channels	6. Cardioprotection	Clements et al. (2015), Testai et al. (2015), Smith et al. (2017), Szteyn and Singh (2020), Lukowski et al. (2021), Pereira and Kowaltowski (2021)
	7. Neuroprotection	Honrath et al. (2017)
	8. Cell death/oncological target	Lang and Hoffmann (2012), Leanza et al. (2014), Leanza et al. (2015), Szabo and Leanza (2017), Leanza et al. (2019), Prosdocimi et al. (2019), Checchetto et al. (2021)
Methods used in mitochondrial potassium channel research	9. Methods of mitochondrial potassium channels measurements	Walewska et al. (2022b)
Regulation of mitochondrial potassium channels	10. Signaling pathways affecting mitochondrial potassium channels	Szabo et al. (2012), Leanza et al. (2013), Leanza et al. (2014), von Charpuis et al. (2015), Rotko et al. (2020b), Kulawiak et al. (2021)
	11. Reactive oxygen species and mitochondrial potassium channels	Trono et al. (2015), O-Uchi et al. (2014)
	12. Gasotransmitters action on mitochondrial potassium channels	Walewska et al. (2018b)
Pharmacology of mitochondrial potassium channels	13. General information on interaction of various drugs with mitochondrial potassium channels	Augustynek et al. (2017), Citi et al. (2018), Leanza et al. (2019), Peruzzo et al. (2020), Wrzosek et al. (2020)
	14. Mitochondrial potassium channel openers	Augustynek et al. (2017), Jiang et al. (2021)
	15. Mitochondrial potassium channel inhibitors	Augustynek et al. (2017)
	16. Non-specific mitochondrial interaction of potassium channel modulators	Olszewska and Szewczyk (2013), Wrzosek et al. (2022)

**FIGURE 1:**
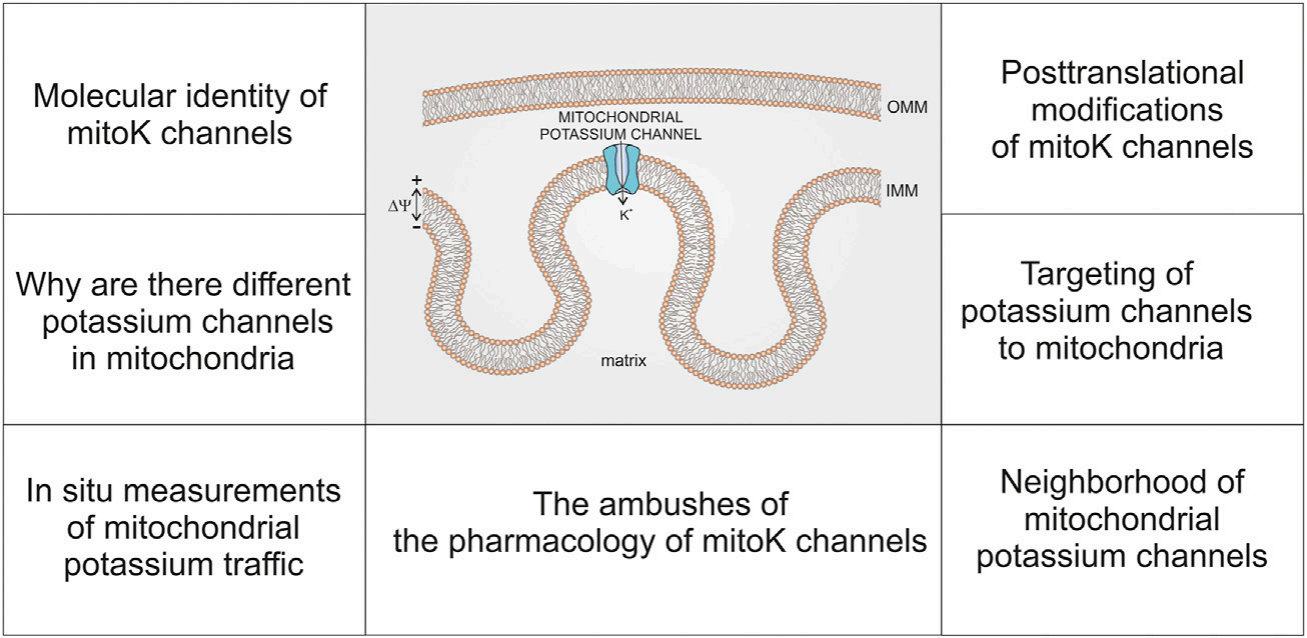
Schematic presentation of topics related to the current state of research on mitochondrial potassium (mitoK) channels—discussed in this report.

In this paper, we will not summarize existing data on mitochondrial potassium channels but rather identify the current challenges of this field (Figure 1). Despite a substantial progress in our understanding of mitochondrial K^+^ traffic, some key issues have not been resolved. Despite advancements in the molecular identification of various mitochondrial potassium channels, there is still no precise map for the distribution of specific potassium channels in various types of mitochondria. Additionally, our understanding of why a simple process of K^+^ influx into mitochondria is catalyzed by so many different channels is not clear. Furthermore, the targeting and import of potassium channels into mitochondria remains a mystery. Moreover, there are strong indications suggesting that interactions of potassium channels with other mitochondrial proteins may create a specific regulatory context in which they function. Understanding the posttranslational modification of mitochondrial potassium channels is still in the early stage of understanding. Another important issue is the development and application of techniques to study the function of mitochondrial potassium channels in intact cells. Finally, the pharmacology of mitochondrial potassium channels, which are very similar to plasma membrane potassium channels, requires careful and thoughtful examination.

We believe that the identification of these challenges may not only be interesting for the reader but will also help to support progress in this field.

### Molecular Identity of Mitochondrial Potassium Channels

For many years, since the discovery of mitoK_ATP_ channels in liver mitochondria (Inoue et al., 1991), mitochondrial potassium channels in various types of tissue cells have been identified on functional bases (Szabo and Zoratti 2014; Laskowski et al., 2016). Mitochondrial potassium channels were simply a phenomenon with no clear molecular origin. It is difficult for some bioenergetics groups to accept the presence of channel-like proteins in the inner mitochondrial membrane. The membrane potential dissipating proteins such as ion channels is not an obvious and obligatory element of chemiosmotic theory (Garlid and Paucek 2003; Jarmuszkiewicz and Szewczyk 2019; Kulawiak et al., 2021; Pereira and Kowaltowski 2021).

Recently, the molecular identity of some mitochondrial potassium channels has been established (Foster et al., 2012; Singh et al., 2013; Paggio et al., 2019; Kulawiak et al., 2021). Based on the biophysical and pharmacological properties, it is believed that the mitochondrial potassium channels have some similarities to plasma membrane potassium channels. Hence, the majority of mitochondrial potassium channels are probably isoforms (splice variants) of plasma membrane potassium channels. Defining the molecular identity of the various mitochondrial potassium channels is one of the most important current challenges in this field. This conclusion also concerns the issue of auxiliary proteins such the β subunits of mitoBK_Ca_ channels or sulfonylurea receptor (SUR) (Szabo and Zoratti 2014; Paggio et al., 2019; Szteyn and Singh 2020).

Initial findings suggested that the mitoK_ATP_ channel was formed by the inward rectifier Kir6.1 or Kir6.2 subunits similar to the plasma membrane K_ATP_ channel. This hypothesis is not supported by current observations. Later studies showed that the ROMK2 potassium channel isoform of the renal outer medullary potassium channel could be the structural component of the mitochondrial channel (Foster et al., 2012; Bednarczyk et al., 2018; Laskowski et al., 2019). Recently, it was shown that the pore-forming subunit of the mitoK_ATP_ channel is a product of the CCDC51 gene (Paggio et al., 2019). The mitoK_ATP_ is inhibited by the antidiabetic sulfonylurea glibenclamide. Therefore, it was speculated that the glibenclamide receptor (SUR), is an integral part of the mitochondrial channel. Indeed, CCDC51 interacts with mitochondrial SUR, encoded by the ABCB8 gene. The mitoK_ATP_ channel formed by these two proteins has the established pharmacological properties of the mitoK_ATP_ channel (Paggio et al., 2019). Interestingly, it was also suggested that the subunits of ATP synthase or some components of the respiratory chain could form mitoK_ATP_ channels. Surprisingly, this channel was sensitive to mitoK_ATP_ channel modulators and F_O_ inhibitors (Juhaszova et al., 2022a; Juhaszova et al., 2022b). It was concluded that mammalian F_1_F_O_ can operate utilizing K^+^ flux and that both K^+^ and H^+^ travel the same route within ATP synthase (Juhaszova et al., 2022a). It cannot be ruled out that ATP-sensitive K^+^ flux in mitochondria may be catalyzed by several different proteins.

The mitoBK_Ca_ channel activity was originally identified in the mitochondria of LN229 glioma cells by the patch-clamp technique (Siemen et al., 1999). Later, similar channel was described in other tissues, including the brain, heart, skeletal muscle, dermal fibroblasts, pulmonary and kidney epithelial cells and endothelium (see references in Table 1). The KCNMA1 gene encodes the pore-forming α subunit of both the mitoBK_Ca_ and the plasma membrane BK_Ca_ channels. The VEDEC isoform of the α subunit is targeted to cardiac mitochondria (Singh et al., 2013). Transfection with a VEDEC-encoding plasmid results in mitoBK_Ca_ channel activity in HEK293T cells (Galecka et al., 2021). Additionally, the functional heterogeneity of mitoBK_Ca_ suggested that there are few mitochondrial isoforms of the α subunit and various auxiliary subunits such as β proteins. Similar channels, have been identified in the mitochondria of lower animals and plants. However, molecular identity of these channels is unclear (Koszela-Piotrowska et al., 2009; Pastore et al., 2013; Laskowski et al., 2015).

The molecular identity of mitochondrial intermediate (mitoIK_Ca_) and small conductance (mitoSK_Ca_) channels is also unclear. Intermediate conductance calcium-activated potassium channels from the mitochondrial inner membrane were identified for the first time in human colon cancer cells. These channels show a conductance close to 27 pS (De Marchi et al., 2009; Dolga et al., 2013; Szabo and Zoratti 2014; Krabbendam et al., 2018; Rotko et al., 2020b). Mitochondrial channels are likely splice variants of plasma membrane channel. The activity of IK_Ca_ channels in the mitochondria and plasma membrane influences oxidative phosphorylation (Kovalenko et al., 2016). In the inner mitochondrial membrane channel with conductance 4–14 pS i.e. small conductance calcium-activated potassium channels have also been identified (Dolga et al., 2013; Stowe et al., 2013; Szabo and Zoratti 2014; Krabbendam et al., 2018; Rotko et al., 2020b).

Few voltage-gated potassium channels, including mitoKv1.3, mitoKv1.5, and mitoKv7.4, have been identified in mitochondria. These channels are similar to those found in the plasma membrane of various cells (Szabo et al., 2005; Checchetto et al., 2018; Wrzosek et al., 2022). The pore-forming α subunit of the Kv1.3 channel contains voltage-sensing domains and is encoded by the KCNA3 gene. Activity of these channels is regulated by auxiliary β subunits. The presence of the mitoKv1.3 channels was observed in T cells and in cancer cells such as leukemia Jurkat T cells, prostate cancer PC-3 cells, B-cell chronic lymphocytic leukemia (CLL), and breast cancer MCF-7 cells (Szabo et al., 2008; Severin et al., 2022). Similar channel have been identified in gerbil hippocampal mitochondria (Bednarczyk et al., 2010). The Kv7 channel subfamily consists of five members named sequentially from Kv7.1 to Kv7.5, encoded by the KCNQ1-5 genes. Each of these channels forms homotetramers, or heterotetramers, that exhibit different tissue distributions and physiological roles. The presence of mitoKv7.4 channels was demonstrated in the H9c2 cardiomyoblast cell line and in adult cardiomyocytes (Testai et al., 2016). Recently the same channel was described in neuronal cells (Paventi et al., 2022).

In the mitochondria of cardiac tissue sodium-activated potassium channels have been identified. These channels belong to the Slo2 family (Wojtovich et al., 2011; Kulawiak et al., 2021). Two genes encode Slo2 channels in mammalian cells: KCNT2, which encodes Slo2.1 (also known as Slick) channels, and KCNT1, which encodes Slo2.2 (Slack) channels (Wojtovich et al., 2011; Kulawiak et al., 2021).

The mammalian TASK-3 channel encoded by KCNK9 gene has been identified in the inner mitochondrial membrane of melanocytes, melanomas (WM35 and B16F10), and keratinocytes. In the human keratinocyte HaCaT cell line the mitochondrial TASK-3 had a conductance of 83 pS at positive voltages and 12 pS at negative voltages in symmetric 150 mM KCl, as measured by the single-channel patch clamp technique (Nagy et al., 2014; Toczylowska-Maminska et al., 2014; Wrzosek et al., 2022).

Hyperpolarization-activated cyclic nucleotide-gated (HCN) channels are localized mainly in the plasma membrane, although they have also been detected in the mitochondria. This was suggested for rat kidneys, human HEK 293 cells, and human cardiac tissue (Leon-Aparicio et al., 2019; Wrzosek et al., 2022). We believe that their presence in mitochondrial membranes and molecular origin need further confirmation.

A very interesting hypothesis has recently been put forward by Zemel and others. Their work suggests existence of nonselective monovalent cation (including K^+^) channel which is closely associated with respiratory chain complex I (Zemel et al., 2022). However, it seems that this issue requires further research.

All of the above information suggests that determining the molecular identity of mitochondrial potassium channels is a very complex and difficult task. We believe this complexity is also due to the dynamic nature of the mitochondria. The structural and functional dynamic nature of mitochondria may lead to heterogeneity of mitochondrial potassium channels expression within the cell. In other words, the mitochondria in a cell may contain different sets of potassium channels. Insufficient information on the molecular identity of mitochondrial potassium channels significantly limits the progress in understanding their functional role and pharmacological properties.

### Why Are There Different Potassium Channels in the Mitochondria

Potassium cation transport in mitochondria plays an important role in mitochondrial volume homeostasis. The complex coupling of mitochondrial matrix swelling and contraction to mitochondrial metabolism was previously described. Additionally, K^+^ transport modulates ROS synthesis in mitochondria (Garlid and Paucek 2003; Jarmuszkiewicz and Szewczyk 2019; Pereira and Kowaltowski 2021). For many years the description of the mitochondrial potassium cycle involved a pathway of K^+^ influx known as potassium uniport. It was assumed that this kind of specific transport activity was catalyzed by one type of protein. Currently, we believe that a set of mitochondrial potassium channels form a pathway (known previously as potassium uniport) of K^+^ influx into the negatively charged mitochondrial matrix (Augustynek et al., 2017; Kulawiak et al., 2021).

As mentioned above, a few different potassium channels have been described in the inner mitochondrial membrane. Why does the inner mitochondrial membrane contain a few various potassium ion channels? Why is a simple process of K^+^ influx not catalyzed by only one type of potassium channel? The way in which this question is answered does testify to our understanding of the functional role of potassium channels in mitochondria. Specifically, this demonstrates our understanding of the physiological role of mitochondrial potassium channels in processes such as ischemia/reperfusion and oxidative stress.

A very good example of the many different routes of potassium ion influx through the inner mitochondrial membrane is the cardiomyocyte mitochondria (Kulawiak et al., 2021). In cardiac mitochondria, the following mitochondrial potassium channels have been identified: mitoK_ATP_ channels, mitoBK_Ca_ channels, mitoSK_Ca_ channels, mitoSlo2 channels, mitoHCN channels and mitochondrial voltage-regulated potassium (mitoKv7.4) channels (Szteyn and Singh 2020; Kulawiak et al., 2021). What is the physiological benefit of using many different ligands and factors to regulate channels? Without experimental data, we speculate that temporal changes in cellular conditions will help us to understand the protective role of various mitochondrial potassium channels.

It is likely that potassium channels present in cardiomyocyte mitochondria are activated under specific circumstances. An early event during cardiac ischemia is ATP depletion, which should lead to mitoK_ATP_ activation. This is followed by mitochondrial membrane depolarization, which probably causes mitoKv7.4 activation (Giorgio et al., 2018; Kulawiak et al., 2021). Moreover, because of ATP depletion, ion pumps cannot function, leading an increase in the cellular Ca^2+^ concentration. The rise in Ca^2+^ during ischemia and reperfusion leads to an overload of mitochondrial Ca^2+^, particularly during reperfusion when oxygen is reintroduced (Borutaite et al., 2013; Giorgio et al., 2018). This should stimulate Ca^2+^-activated potassium channels such as mitoSK_Ca_ channels and mitoBK_Ca_ channels. During ischemia, anaerobic glycolysis, ATP hydrolysis, and the release of protons from acidic organelles cause the pH to decrease by approximately one unit, affecting mitoK_ATP_ activity. The decrease in intracellular pH during severe ischemia promotes the imbalance of other cations, leading to cellular Na^+^ overload (Borutaite et al., 2013; Kulawiak et al., 2021). This process may lead to Na^+^-activated mitoSlo2 channel stimulation. Reintroduction of oxygen during reperfusion allows synthesis of ATP and mitoK_ATP_ inhibition. However, restarting the electron transport chain results in increase of mitochondrial generation of reactive oxygen species (ROS). An increase in ROS may stimulate mitoK_ATP_ and mitoBK_Ca_ channels (Rotko et al., 2020b; Kulawiak et al., 2021).

This complex view of channel activation/inhibition possibly explains why there are few potassium channels. Most likely, the timing of changes such as ATP, pH, calcium and sodium cation concentration changes is critical to control K^+^ flux in mitochondria. *In situ* measurements of specific channel activity would help to identify when it becomes activated. However, how can the activity of intracellular K^+^ channels be measured in intact cells?

### *In situ* Measurements of Mitochondrial K^+^ Traffic

The majority of techniques to study mitochondrial K^+^ transport were previously developed to characterize plasma membrane potassium channels. Most likely, measuring the mitochondrial swelling is the only unique technique that can follow the influx of K^+^ into mitochondria (Pereira and Kowaltowski 2021; Walewska et al., 2022b). Unfortunately, this technique cannot distinguish K^+^ flux via specific mitochondrial potassium channels. Moreover, the density of some mitochondrial channels is very low (Laskowski et al., 2016; Pereira and Kowaltowski 2021; Walewska et al., 2022b). Application of pharmacological tools is not helping to use mitochondrial swelling in mitochondrial channel studies. Hence, establishing technique for functional *in situ* studies of mitochondrial potassium channels is a real challenge.

The main difficulty in studying mitochondrial potassium channels is the need for isolating mitochondria and the mitochondrial inner membrane before functional experiments. This means that researchers must precisely design experiments to ensure that mitochondrial potassium channels, rather than plasma membrane channels, are studied in preparation. The reliability of functional studies on mitochondrial potassium channels is additionally influenced by the functional properties of mitochondria, such as their respiration, metabolic activity, swelling capacity or high electrical potential.

Key techniques used in mitochondrial potassium channel studies include i. electrophysiological methods (patch-clamp and planar lipid bilayer), ii. direct potassium flux measurements, and iii. biochemical techniques that are indirectly related to potassium movement across the inner mitochondrial membrane (Walewska et al., 2022b).

Key knowledge about the biophysical properties of the potassium channels (such as conductance or selectivity) was obtained with the application of the planar lipid bilayer technique and patch-clamp technique (Szabo and Zoratti 2014; Walewska et al., 2022b). These techniques are well established and widely used for studies of ion channels from the plasma membrane. However, the application of both techniques for mitochondrial potassium channel experiments requires a special approach. The first challenge is the need to use a membrane fraction with a very high purity. Due to the location of potassium channels in various organelles and cell compartments, this step is particularly important when using planar lipid membranes. These techniques not only helped to measure biophysical properties of the channels but were also crucial for identifying the pharmacology of mitochondrial potassium channels. Both electrophysiological techniques are demanding and require appropriate sample preparation and experience.

Direct measurement of K^+^ flux across the inner membrane is also possible by applying of fluorescent probes, i.e., small molecule probes, genetically encoded probes, and thallium (Tl^+^)-sensitive indicators (Walewska et al., 2022b). All these techniques are not easy to apply to study mitochondrial potassium channels in intact cells. Developing genetically encoded specific probes is likely a promising but not easy direction. One of the most interesting tools is the genetically encoded potassium ion indicator (GEPII) based on Förster resonance energy transfer-(FRET) (Bischof et al., 2017). The probe may be targeted to mitochondria and other intracellular compartments (Bischof et al., 2017). However, influx of potassium in mitochondrial matrix is accompanied by matrix volume increase that leads to complex interpretation of experiments.

Finally, the application of biochemical techniques due to unique mitochondrial activity allows the changes in mitochondrial function induced by mitochondrial potassium activity to be monitored. This approach includes measurements of respiration and membrane potential to track potassium fluxes via inner membrane channel proteins. Because mitochondrial respiration and potential depend on various conditions K^+^ traffic via channels is not simple (Walewska et al., 2022b).

The contribution of all these methods to advancing our knowledge on mitochondrial K^+^ channels are important. However, a good and easy technique to measure mitochondrial potassium channels in intact cells is expected in the future. This kind of approach would progress our understanding of the functional role of mitochondrial potassium channels.

### The Ambushes of the Pharmacology of Mitochondrial Potassium Channels

An important part of mitochondrial potassium channel identification is based on the usage of specific chemical substances. The application of pharmacological modulators of mitochondrial potassium channels is crucial for the characterization of channel proteins (Szabo and Zoratti 2014; Leanza et al., 2019; Wrzosek et al., 2020). The majority of these modulators were previously used to characterize the potassium channels present in the plasma membrane. The group of modulators includes channel activators known as potassium channel openers and channel inhibitors such as blockers. Among both groups of modulators, there are synthetic substances and substances of natural origin, such as flavonoids or neurotoxins (Wrzosek et al., 2020). The application of these substances has been essential for the identification of mitochondrial potassium channels in mitochondria by electrophysiological techniques that focus on single-channel activities (Szabo and Zoratti 2014; Laskowski et al., 2016; Leanza et al., 2019).

However, experiments characterizing the role of mitochondrial potassium channels in isolated mitochondria or in intact cells revealed that pharmacological tools have serious limitations. Unfortunately, the majority of mitochondrial potassium channel modulators exhibit a wide range of off-target effects (Szabo et al., 2021; Wrzosek et al., 2022). Mitochondria seem to be especially susceptible to the nonspecific action of channel modulators. This action includes uncoupling properties, respiratory chain inhibition, and effects on cellular calcium homeostasis (Augustynek et al., 2017; Wrzosek et al., 2020). Therefore, the correct application of potassium channel inhibitors or activators on mitochondrial potassium channels is crucial in functional studies on intact cells. Since the discovery of potassium channels in eukaryotic cells, a large number of endogenous and synthesized substances have been discovered that modulate potassium channel activity. Due to the similar structure of potassium channels, some of these compounds interact with the channels found in the inner mitochondrial membrane. As mentioned above, a number of plasma membrane modulators, potassium channel openers, and inhibitors have been tested, and some have also been shown to regulate potassium channels that are located in the inner mitochondrial membrane (Leanza et al., 2019; Wrzosek et al., 2020; Wrzosek et al., 2022).

Unfortunately, the accumulation of drugs in mitochondria increases the probability of side effects (off-target effects) on mitochondrial enzymes, especially interactions with the respiratory chain or ATP synthase, which may be harmful due to multiple negative consequences on cellular function (Leanza et al., 2019; Wrzosek et al., 2020; Wrzosek et al., 2022).

The scales of difficulty are illustrated by, for example, the use of a diazoxide potassium channel activator. Diazoxide is still used as the primary treatment to control hypoglycemia in insulinoma (Wrzosek et al., 2022). Interestingly, mitoK_ATP_ channel is more sensitive to diazoxide than its counterpart in the plasma membrane. It was also observed that diazoxide is responsible for protecting heart cells against ischemia and reperfusion heart injury. However, this compound has also protonophoretic (uncoupling) properties and inhibits mitochondrial succinate dehydrogenase. There seems to be a debate as to whether the cytoprotective effect of diazoxide is due to mitoK_ATP_ activation or if it acts synergistically with other targets (Leanza et al., 2019; Wrzosek et al., 2020). Cytoprotection induced by diazoxide might be a result of complex II inhibition and production of reactive oxygen species and mitoK_ATP_ channels may be involved in cytoprotection as an independent factor (Leanza et al., 2019; Wrzosek et al., 2020). It has been speculated that by targeting nucleotide-requiring enzymes, particularly SDH and cellular ATPases, diazoxide reduces ROS generation and nucleotide degradation, resulting in the preservation of ATP levels in tissue during ischemia.

Moreover, properties of mitochondria such as high membrane potential, alkaline matrix and presence of respiratory chain generating protonmotive force are not helping for finding specific modulators of mitochondrial potassium channels. The identification of potassium channel modulators that are specific for mitochondrial potassium channels is an important aim of future studies. It is important for more than improving the identification of mitochondrial potassium channels. Specific drugs acting only on mitochondrial potassium channels in specific tissues or cell types will accelerate the use of these substances in medical applications, such as cytoprotection of ischemic tissue.

### Neighborhood of Mitochondrial Potassium Channels

The environment in which mitochondrial potassium channels operate differs from that of the plasma membrane. This unique environment mainly involves a unique set of proteins in mitochondria, some of which interact directly with channel proteins (Kathiresan et al., 2009; Singh et al., 2016; Rotko et al., 2020b). Many of them are involved in redox reactions (Singh et al., 2016). Additionally, the active role of mitochondria as the site of ROS synthesis differs considerably from that of the plasma membrane. Mitochondrial potassium channels are regulated by factors such as ATP and Ca^2+^, which are important participants in mitochondrial metabolism (Szabo and Zoratti 2014; Rotko et al., 2020b).

The structural-functional interactions of mitochondrial potassium channels with respiratory chain proteins exhibits a strong context of channel functioning in mitochondria. A unique (specific for mitochondria) regulation of mitochondrial potassium channels was reported (Bednarczyk et al., 2013). The single-channel activity of the mitoBK_Ca_ channel was measured by patch-clamping mitoplasts isolated from the human astrocytoma (glioblastoma) U-87 MG cell line (Bednarczyk et al., 2013). It was shown that substrates of the respiratory chain, such as NADH, succinate, and glutamate/malate, decrease the activity of the channel. This effect was abolished by rotenone, antimycin, and cyanide, which are inhibitors of the mitochondrial respiratory chain. The putative interaction of the β4 subunit of mitoBK_Ca_ with cytochrome *c* oxidase (COX) was demonstrated using the blue native electrophoresis technique. These results indicated possible structural and functional coupling of the mitoBK_Ca_ channel with the mitochondrial respiratory chain in human astrocytoma U-87 MG cells (Bednarczyk et al., 2013). Directly regulating mitoBK_Ca_ channels by mitochondrial respiratory chain redox status may play an important role in the ischemia reperfusion phenomenon. In addition, a physical interaction was observed between the mitochondrial respiratory chain complex I and the mitoKv1.3 channel (Peruzzo et al., 2020).

The interaction of the mitoBK_Ca_ channel with COX has an additional interesting functional consequences. It is known that mitochondrial respiratory chain absorb near-infrared light between 700 and 1400 nm. In the near-infrared region, the 820 nm absorption band belongs mainly to the relatively oxidized CuA and the 760 nm absorption band to the relatively reduced CuB chromophore of COX. The absorption of infrared light by COX enhances respiratory chain function and increase the synthesis of ATP by mitochondria. The mitoBK_Ca_ channels of the astrocytoma cell line was investigated using a patch clamp technique with an illumination system (Szewczyk and Bednarczyk 2018). It was found that the mitoBK_Ca_ channel activity was modulated by illumination by infrared light. Activation of the mitoBK_Ca_ channel was observed after illumination using specific infrared light wavelengths. These findings confirmed the functional coupling of the respiratory chain via COX to the mitoBK_Ca_ channel and regulation its activity by infrared light (Szewczyk and Bednarczyk 2018).

Mitochondria are highly dynamic intracellular structures in which inner mitochondrial membrane can be dramatically remodeled, depending on metabolic activity. It was shown for the first time that mechanical stimulation of the mitoBK_Ca_ channel resulted in an increased probability of channel opening as measured by the patch-clamp technique in mitochondria isolated from human astrocytoma U-87 MG cells (Walewska et al., 2018a). These results indicated the possible involvement of mitoBK_Ca_ channels in mitochondrial activities in which changes in membrane shape and tension play a crucial role, such as fusion/fission and cristae remodeling (Walewska et al., 2018b).

These examples illustrate that localization of potassium channels in the mitochondrial inner membrane may form a new context of channel functioning and regulation. Understanding the new channel regulatory mechanisms, as a consequence of mitochondrial localization, seems to be an important challenge in the field of mitochondrial potassium channels.

### Targeting of Potassium Channels to Mitochondria

Mitochondria have their own genome, which makes them semiautonomous organelles. However, mitochondrial DNA encodes only a small part of the mitochondrial proteome. Genes encoding most mitochondrial proteins are located in the nucleus and are translated by cytosolic ribosomes. Therefore, more than 99% of mitochondrial proteins must be imported into mitochondria. The same is true for the genes that encode mitochondrial potassium channels (Wiedemann and Pfanner 2017). However, little is known about the mechanism responsible for targeting potassium channels to mitochondria.

Many mitochondrial proteins translated in the cytosol have mitochondrial targeting signals (Chacinska et al., 2009; Wiedemann and Pfanner 2017). This signal can be localized in a specific region (e.g., presequence in the N-terminal part of the protein) or can be dispersed in different parts of the protein. Mitochondria are equipped in the system of protein translocases that recognize and sort imported proteins to mitochondrial compartments such as the matrix, inner mitochondrial membrane, intramembrane space, and mitochondrial outer membrane. Important steps of mitochondrial protein maturation may include cleavage of the mitochondrial presequence by mitochondrial proteases (Kulawiak et al., 2013; Wiedemann and Pfanner 2017).

Mitochondrial potassium channels are a heterogeneous group of proteins and include proteins with two transmembrane segments (such as ROMK protein or regulatory β subunits of mitoBK_Ca_ channels) and multispanning proteins (such as pore forming α subunit of the mitoBK_Ca_ or mitoKv1.3 channels).

Interestingly, the only mitochondrial potassium channel localized exclusively in the mitochondrial inner membrane is the CCDC51 protein. This protein has been identified as the pore-forming protein of the mitoK_ATP_ channel (Paggio et al., 2019). The amino acid sequence of CCDC51 suggests the existence of two transmembrane segments which makes it a good candidate for a TIM23 substrate. Similarly, ROMK2 protein also has two transmembrane segments. Interestingly, it has been suggested that this isoform has a mitochondrial targeting sequence localized in the N-terminus, which also makes it a potential substrate for TIM23 translocase (Foster et al., 2012). On the other hand, in the case of the mitoBK_Ca_ channel, the situation is probably different. The channel pore-forming α subunit has several transmembrane domains, which probably means that import to mitochondria arises a different path (Szteyn and Singh, 2020). However, a recent study showed that mitochondrial import of the Kv1.3 channel (which is a multispanning protein) is mediated by TIM23 translocase (Capera et al., 2022). This unexpected observation points to the need for a more detailed study of this issue.

**FIGURE 2:**
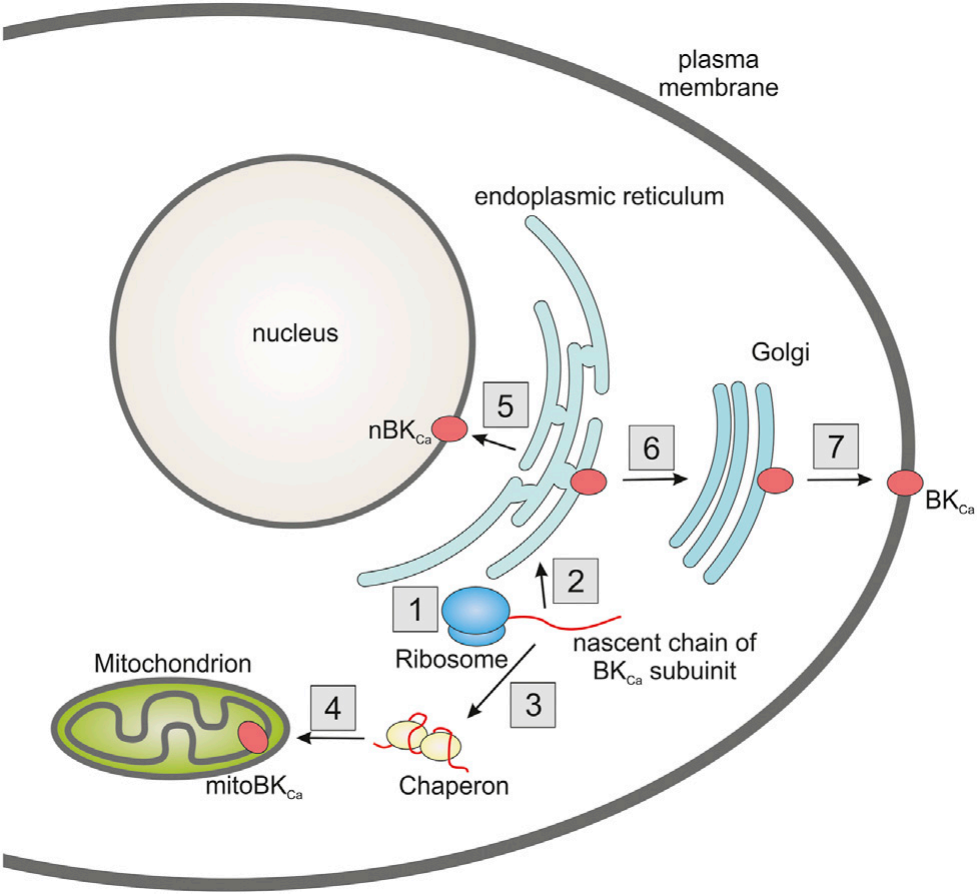
Diagram showing the problem of targeting potassium channels to multiple cell compartments using the example of a BK_Ca_ channel. The nascent pore forming subunit of the channel (1) can be targeted to the endoplasmic reticulum as its primary destination (2). A certain fraction of the protein, possibly using chaperones (3), can be directed to the mitochondria (4). From the endoplasmic reticulum, protein can be directed to other cell compartments such as the nucleus (5) or via the Golgi apparatus (6) to the plasma membrane (7). Probably the target location of the protein may also be influenced by the translation rate, which may be influenced by the codon usage bias (1). In addition, an important role in protein targeting may be played by alternative splicing, which results in the formation of various isoforms containing the appropriate targeting sequences.

Another issue is sorting of the mitochondrial potassium channel subunits between various cellular compartments. For example, BK_Ca_ channels are found in the plasma membrane, nucleus or mitochondria (Figure 2). This requires the existence of an efficient protein distribution system. Regarding the BK_Ca_ channel, it is possible that, in some cases, selection may be accomplished by mRNA splicing. For example, in cardiomyocytes it has been shown that the VEDEC isoform of α subunit is exclusively targeted to mitochondria (Singh et al., 2013). It is also possible that chaperone proteins play an important role in the distribution mechanism. HSP60 has been shown to interact with the α subunit of the BK_Ca_ channel. It has been proposed that this interaction might increase mitochondrial targeting of this subunit (Singh et al., 2016).

Another interesting observation is that codon bias can alter the sorting of virus potassium channels between mitochondria and other cellular compartments (Engel et al., 2021). It has been described that codon optimization results in increased sorting of viral potassium channels to mitochondria. This seemed to be valid for several cell lines which suggests a general mechanism (Engel et al., 2021). Therefore, this line of research seems very interesting, especially when considering that different potassium channels may be present in the mitochondria of different tissues.

### Posttranslational Modifications of Mitochondrial Potassium Channels

Posttranslational modifications (PTMs) of membrane proteins, including ion channels, are one of the most important regulatory mechanisms of protein function and activity (Stram and Payne 2016; Aranda-Rivera et al., 2021). The issue of PTMs of mitochondrial potassium channels is a broad topic and the experimental data available thus far is limited. Therefore, detailed analysis of posttranslational modifications of mitochondrial potassium channels is still one of the main challenges in the field.

**FIGURE 3:**
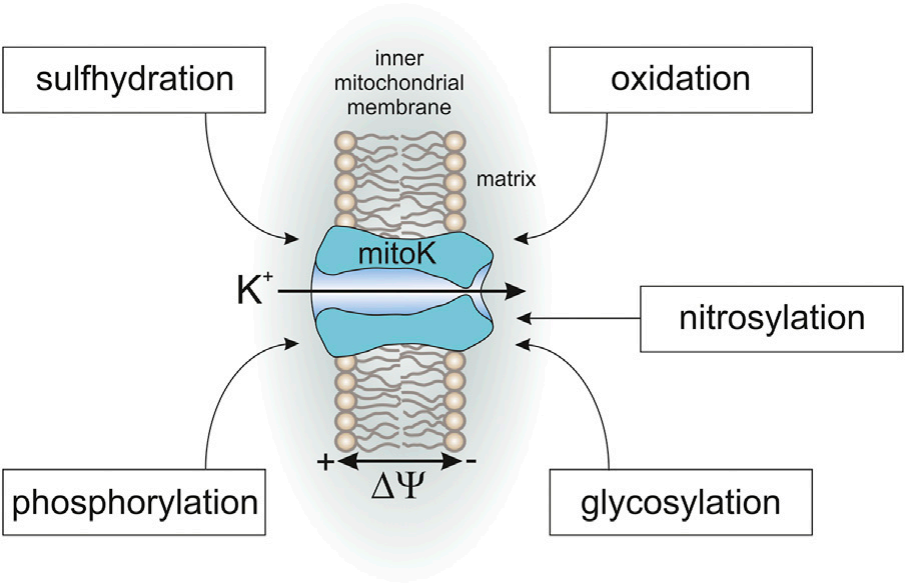
Posttranslational modifications of mitochondrial potassium channels.

Many studies have described the phosphorylation of mitochondria potassium channels is as an important step in the cardioprotection mechanism in murine, rabbit, canine and *in vitro* cell culture models. It has been found that protein kinase C, protein kinase A and protein kinase G regulate the activity of selected mitochondrial potassium channels, including mitoK_ATP_ and mitoBK_Ca_ (Figure 3) (Rotko et al., 2020b; Checchetto et al., 2021). Apparently, the phosphorylation of mitochondrial potassium channels might be a trigger for the induction of a cardioprotective cascade. Despite numerous data showing that phosphorylation affects channel activity and is important in cytoprotection, there is still little data on phosphorylation sites or the relationship between the signaling pathways that lead to cytoprotection. This thread is of particular interest considering the presence of several potassium channels in the mitochondria from the same tissue.

Another example of PTM is directly related to the regulation of mitochondrial potassium channel activity by gaseous transmitters such as carbon monoxide (CO), hydrogen sulfide (H_2_S) or nitric oxide (NO) (Walewska et al., 2018b; Rotko et al., 2020a). Regulation of mitochondrial potassium channels activity by these gases is directly connected with posttranslational modifications such as S-nitrosylation or S-sulfhydration. However, the influence of this type of PTM on mitochondrial potassium channels is more complex. For example, a recent study showed that applications of the H_2_S donor NaHS result in the mitoBK_Ca_ S-sulfhydration of cysteines; however, this did not change the channel activity (Walewska et al., 2022a). The regulation of activity by gases is also related to the interaction of cofactors with channels such as heme (Walewska et al., 2018b; Rotko et al., 2020a; Rotko et al., 2020b). Nevertheless, the regulation of channel activity by gases seems to be important from the perspective of cytoprotective mechanisms, and a detailed understanding of this regulatory mechanism should be an important direction of research.

It seems that redox reactions play an important role in the cytoprotective mechanism. Mitochondrial potassium channels can act as redox sensors and are targets for reactive oxygen species, which may induce post-translational modifications (Aranda-Rivera et al., 2021; Kulawiak et al., 2021; Queliconi et al., 2011; O-Uchi et al., 2014; Trono et al., 2015). It has been shown that this may be necessary for the activation of cardioprotection (Queliconi et al., 2011; Rotko et al., 2020b). On the other hand, channel activation may regulate the synthesis of reactive oxygen species in the mitochondria. Regulation of the synthesis of reactive oxygen species also appears to be an important element of the cytoprotective signaling pathway (Rotko et al., 2020b; Borchert et al., 2013; O-Uchi et al., 2014; Trono et al., 2015).

Another potential and interesting issue is the glycosylation of mitochondrial channel subunits. It is known that the β regulatory subunits of plasma membrane BK_Ca_ channels are N-glycosylated. This has a major impact on the properties and regulation of the BK_Ca_ channels (Meera et al., 2000; Martinez-Espinosa et al., 2014). However, N-glycosylation only seems to occur outside the mitochondria. On the other hand, another type of glycosylation such as *O*-GlcNAcylation takes place in mitochondria, which may be an indication for the direction of research in this area. For example, this type of modification has been described for VDAC (Johnsen et al., 2013; Zhao et al., 2016). However, the fact that *O*-GlcNAcylation is a more dynamic process must be taken into account, and this causes some technical problems in regard to the methodological approach to this research (Zhao et al., 2016).

### Final Remarks

In this paper, we have defined a few current challenges of mitochondrial potassium channel research. Despite 30 years of studies and substantial progress in understanding K^+^ traffic through the mitochondrial inner membrane, still some essential problems remain unresolved. Most likely, the most important issue is the identification of the molecular identity of mitochondrial potassium channels. Due to progress in this area, advancements in understanding the interactions of potassium channel proteins with other mitochondrial proteins and identifying of specific pharmacology, etc., will be possible. Progress in the techniques of measuring mitochondrial potassium channels will help to clarify of the physiological role of these proteins. We hope that clearly expressing all these problems will help to advance the field of mitochondrial potassium channels.

## References

[B1] Aranda-Rivera A. K. Cruz-Gregorio A. Aparicio-Trejo O. E. Ortega-Lozano A. J. Pedraza-Chaverri J. (2021). Redox Signaling Pathways in Unilateral Ureteral Obstruction (UUO)-induced Renal Fibrosis. Free Radic. Biol. Med. 172, 65–81. 10.1016/j.freeradbiomed.2021.05.034 34077780

[B2] Augustynek B. Kunz W. S. Szewczyk A. (2017). Guide to the Pharmacology of Mitochondrial Potassium Channels. Handb. Exp. Pharmacol. 240, 103–127. 10.1007/164_2016_79 27838853

[B3] Bachmann M. Li W. Edwards M. J. Ahmad S. A. Patel S. Szabo I. (2020). Voltage-Gated Potassium Channels as Regulators of Cell Death. Front. Cell Dev. Biol. 8, 611853. 10.3389/fcell.2020.611853 33381507 PMC7767978

[B4] Balderas E. Zhang J. Stefani E. Toro L. (2015). Mitochondrial BKCa Channel. Front. Physiol. 6, 104. 10.3389/fphys.2015.00104 25873902 PMC4379900

[B5] Bednarczyk P. Kicinska A. Laskowski M. Kulawiak B. Kampa R. Walewska A. (2018). Evidence for a Mitochondrial ATP-Regulated Potassium Channel in Human Dermal Fibroblasts. Biochim. Biophy. Acta. Bioenerg. 1859, 309–318. 10.1016/j.bbabio.2018.02.005 29458000

[B6] Bednarczyk P. Kowalczyk J. E. Beręsewicz M. Dołowy K. Szewczyk A. Zabłocka B. (2010). Identification of a Voltage-Gated Potassium Channel in Gerbil Hippocampal Mitochondria. Biochem. Biophys. Res. Commun. 397, 614–620. 10.1016/j.bbrc.2010.06.011 20570656

[B7] Bednarczyk P. Wieckowski M. R. Broszkiewicz M. Skowronek K. Siemen D. Szewczyk A. (2013). Putative Structural and Functional Coupling of the Mitochondrial BKCa Channel to the Respiratory Chain. PLoS One 8, e68125. 10.1371/journal.pone.0068125 23826369 PMC3694950

[B8] Bischof H. Rehberg M. Stryeck S. Artinger K. Eroglu E. Waldeck-Weiermair M. (2017). Novel Genetically Encoded Fluorescent Probes Enable Real-Time Detection of Potassium *In Vitro* and *In Vivo* . Nat. Commun. 8, 1422. 10.1038/s41467-017-01615-z 29127288 PMC5681659

[B9] Borchert G. H. Hlaváčková M. Kolář F. (2013). Pharmacological Activation of Mitochondrial BKCa Channels Protects Isolated Cardiomyocytes against Simulated Reperfusion-Induced Injury. Exp. Biol. Med. 238, 233–241. 10.1177/1535370212474596 23576804

[B10] Borutaite V. Toleikis A. Brown G. C. (2013). In the Eye of the Storm: Mitochondrial Damage during Heart and Brain Ischaemia. FEBS J. 280, 4999–5014. 10.1111/febs.12353 23710974

[B11] Capera J. Navarro-Pérez M. Moen A. S. Szabó I. Felipe A. (2022). The Mitochondrial Routing of the Kv1.3 Channel. Front. Oncol. 12, 865686. 10.3389/fonc.2022.865686 35402277 PMC8990977

[B12] Chacinska A. Koehler C. M. Milenkovic D. Lithgow T. Pfanner N. (2009). Importing Mitochondrial Proteins: Machineries and Mechanisms. Cell 138, 628–644. 10.1016/j.cell.2009.08.005 19703392 PMC4099469

[B13] Checchetto V. Azzolini M. Peruzzo R. Capitanio P. Leanza L. (2018). Mitochondrial Potassium Channels in Cell Death. Biochem. Biophys. Res. Commun. 500, 51–58. 10.1016/j.bbrc.2017.06.095 28642134

[B14] Checchetto V. Leanza L. De Stefani D. Rizzuto R. Gulbins E. Szabo I. (2021). Mitochondrial K+ Channels and Their Implications for Disease Mechanisms. Pharmacol. Ther. 227, 107874. 10.1016/j.pharmthera.2021.107874 33930454

[B15] Checchetto V. Teardo E. Carraretto L. Leanza L. Szabo I. (2016). Physiology of Intracellular Potassium Channels: A Unifying Role as Mediators of Counterion Fluxes? Biochimica Biophys. Acta.Bioenerg. 1857, 1258–1266. 10.1016/j.bbabio.2016.03.011 26970213

[B16] Citi V. Calderone V. Martelli A. Breschi M. C. Testai L. (2018). Pathophysiological Role of Mitochondrial Potassium Channels and Their Modulation by Drugs. Cmc 25, 2661–2674. 10.2174/0929867324666171012115300 29022502

[B17] Clements R. T. Terentyev D. Sellke F. W. (2015). Ca2+-Activated K+ Channels as Therapeutic Targets for Myocardial and Vascular Protection. Circ. J. 79, 455–462. 10.1253/circj.cj-15-0015 25746520

[B18] De Marchi U. Sassi N. Fioretti B. Catacuzzeno L. Cereghetti G. M. Szabò I. (2009). Intermediate Conductance Ca2+-Activated Potassium Channel (KCa3.1) in the Inner Mitochondrial Membrane of Human Colon Cancer Cells. Cell Calcium 45, 509–516. 10.1016/j.ceca.2009.03.014 19406468

[B19] Dolga A. M. Netter M. F. Perocchi F. Doti N. Meissner L. Tobaben S. (2013). Mitochondrial Small Conductance SK2 Channels Prevent Glutamate-Induced Oxytosis and Mitochondrial Dysfunction. J. Biol. Chem. 288, 10792–10804. 10.1074/jbc.m113.453522 23430260 PMC3624460

[B20] Engel A. J. Kithil M. Langhans M. Rauh O. Cartolano M. Van Etten J. L. (2021). Codon Bias Can Determine Sorting of a Potassium Channel Protein. Cells, 10, 1128. 10.3390/cells10051128 34066987 PMC8151079

[B21] Foster D. B. Ho A. S. Rucker J. Garlid A. O. Chen L. Sidor A. (2012). Mitochondrial ROMK Channel Is a Molecular Component of MitoK ATP. Circ. Res. 111, 446–454. 10.1161/circresaha.112.266445 22811560 PMC3560389

[B22] Galecka S. Kulawiak B. Bednarczyk P. Singh H. Szewczyk A. (2021). Single Channel Properties of Mitochondrial Large Conductance Potassium Channel Formed by BK-VEDEC Splice Variant. Sci. Rep. 11, 10925. 10.1038/s41598-021-90465-3' 34035423 PMC8149700

[B23] Garlid K. D. Paucek. P. (2003). Mitochondrial Potassium Transport: the K+ Cycle. Biochimica Biophysica. Acta.Bioenerg. 1606, 23–41. 10.1016/s0005-2728(03)00108-7 14507425

[B24] Giorgio V. Guo L. Bassot C. Petronilli V. Bernardi P. (2018). Calcium and Regulation of the Mitochondrial Permeability Transition. Cell Calcium 70, 56–63. 10.1016/j.ceca.2017.05.004 28522037

[B25] González-Sanabria N. Echeverría F. Segura I. Alvarado-Sánchez R. Latorre R. (2021). BK in Double-Membrane Organelles: A Biophysical, Pharmacological, and Functional Survey. Front. Physiol. 12, 761474. 10.3389/fphys.2021.761474 34764886 PMC8577798

[B26] Honrath B. Krabbendam I. E. Culmsee C. Dolga A. M. (2017). Small Conductance Ca 2+ -activated K + Channels in the Plasma Membrane, Mitochondria and the ER: Pharmacology and Implications in Neuronal Diseases. Neurochem. Int. 109, 13–23. 10.1016/j.neuint.2017.05.005 28511953

[B27] Inoue I. Nagase H. Kishi K. Higuti T. (1991). ATP-sensitive K+ Channel in the Mitochondrial Inner Membrane. Nature 352, 244–247. 10.1038/352244a0 1857420

[B28] Jarmuszkiewicz W. Szewczyk A. (2019). Energy-dissipating Hub in Muscle Mitochondria: Potassium Channels and Uncoupling Proteins. Archives Biochem. Biophysics 664, 102–109. 10.1016/j.abb.2019.01.036 30716282

[B29] Jiang X. Wu D. Jiang Z. Ling W. Qian G. (2021). Protective Effect of Nicorandil on Cardiac Microvascular Injury: Role of Mitochondrial Integrity. Oxid. Med. Cell Longev. 2021, 4665632. 10.1155/2021/4665632 34285763 PMC8275446

[B30] Johnsen V. L. Belke D. D. Hughey C. C. Hittel D. S. Hepple R. T. Koch L. G. (2013). Enhanced Cardiac Protein Glycosylation (O-GlcNAc) of Selected Mitochondrial Proteins in Rats Artificially Selected for Low Running Capacity. Physiol. Genomics 45, 17–25. 10.1152/physiolgenomics.00111.2012 23132757 PMC3544485

[B31] Juhaszova M. Kobrinsky E. Zorov D. B. Bradley Nuss H. Yaniv Y. Fishbein K. W. (2022b). ATP Synthase K+- and H+-fluxes Drive ATP Synthesis and Enable Mitochondrial K+-“Uniporter” Function: II. Ion and ATP Synthase Flux Regulation. Funct. (Oxf) 3, zqac001. 10.1093/function/zqac001' PMC8850977 35187492

[B32] Juhaszova M. Kobrinsky E. Zorov D. B. Nuss H. B. Yaniv Y. Fishbein K. W. (2022a). 'ATP Synthase K(+)- and H(+)-Fluxes Drive ATP Synthesis and Enable Mitochondrial K(+)-"Uniporter" Function: I. Characterization of Ion Fluxes. Funct. (Oxf) 3, zqab065. 10.1093/function/zqab065 PMC8867323 35229078

[B33] Kathiresan T. Harvey M. Orchard S. Sakai Y. Sokolowski B. (2009). A Protein Interaction Network for the Large Conductance Ca2+-Activated K+ Channel in the Mouse Cochlea. Mol. Cell. Proteomics 8, 1972–1987. 10.1074/mcp.m800495-mcp200 19423573 PMC2722780

[B34] Koszela-Piotrowska I. Matkovic K. Szewczyk A. Jarmuszkiewicz W. (2009). A Large-Conductance Calcium-Activated Potassium Channel in Potato (Solanum tuberosum) Tuber Mitochondria. Biochem. J. 424, 307–316. 10.1042/bj20090991 19740073

[B35] Kovalenko I. Glasauer A. Schöckel L. Sauter D. R. P. Ehrmann A. Sohler F. (2016). Identification of KCa3.1 Channel as a Novel Regulator of Oxidative Phosphorylation in a Subset of Pancreatic Carcinoma Cell Lines. PLoS One 11, e0160658. 10.1371/journal.pone.0160658 27494181 PMC4975431

[B36] Krabbendam I. E. Honrath B. Culmsee C. Dolga A. M. (2018). Mitochondrial Ca2+-Activated K+ Channels and Their Role in Cell Life and Death Pathways. Cell Calcium 69, 101–111. 10.1016/j.ceca.2017.07.005 28818302

[B37] Kravenska Y. Checchetto V. Szabo I. (2021). Routes for Potassium Ions across Mitochondrial Membranes: A Biophysical Point of View with Special Focus on the ATP-Sensitive K+ Channel. Biomolecules 11, 1172. 10.3390/biom11081172' 34439838 PMC8393992

[B38] Kulawiak B. Bednarczyk P. Szewczyk A. (2021). Multidimensional Regulation of Cardiac Mitochondrial Potassium Channels. Cells 10, 1554. 10.3390/cells10061554 34205420 PMC8235349

[B39] Kulawiak B. Höpker J. Gebert M. Guiard B. Wiedemann N. Gebert N. (2013). The Mitochondrial Protein Import Machinery Has Multiple Connections to the Respiratory Chain. Biochimica Biophys. Acta. Bioenerg. 1827, 612–626. 10.1016/j.bbabio.2012.12.004 23274250

[B40] Lang F. Hoffmann E. K. (2012). Role of Ion Transport in Control of Apoptotic Cell Death. Compr. Physiol. 2, 2037–2061. 10.1002/cphy.c110046 23723032

[B41] Laskowski M. Augustynek B. Bednarczyk P. Żochowska M. Kalisz J. O'Rourke B. (2019). Single-Channel Properties of the ROMK-Pore-Forming Subunit of the Mitochondrial ATP-Sensitive Potassium Channel. Int. J. Mol. Sci. 20, 5323. 10.3390/ijms20215323 PMC6862428 31731540

[B42] Laskowski M. Augustynek B. Kulawiak B. Koprowski P. Bednarczyk P. Jarmuszkiewicz W. (2016). What Do We Not Know about Mitochondrial Potassium Channels? Biochimica Biophys. Acta. Bioenerg. 1857, 1247–1257. 10.1016/j.bbabio.2016.03.007 26951942

[B43] Laskowski M. Kicinska A. Szewczyk A. Jarmuszkiewicz W. (2015). Mitochondrial Large-Conductance Potassium Channel from Dictyostelium discoideum. Int. J. Biochem. Cell Biol. 60, 167–175. 10.1016/j.biocel.2015.01.006 25596489

[B44] Leanza L. Biasutto L. Managò A. Gulbins E. Zoratti M. Szabò I. (2013). Intracellular Ion Channels and Cancer. Front. Physiol. 4, 227. 10.3389/fphys.2013.00227 24027528 PMC3759743

[B45] Leanza L. Checchetto V. Biasutto L. Rossa A. Costa R. Bachmann M. (2019). Pharmacological Modulation of Mitochondrial Ion Channels. Br. J. Pharmacol. 176, 4258–4283. 10.1111/bph.14544 30440086 PMC6887689

[B46] Leanza L. Venturini E. Kadow S. Carpinteiro A. Gulbins E. Becker K. A. (2015). Targeting a Mitochondrial Potassium Channel to Fight Cancer. Cell Calcium 58, 131–138. 10.1016/j.ceca.2014.09.006 25443654

[B47] Leanza L. Zoratti M. Gulbins E. Szabo I. (2014). Mitochondrial Ion Channels as Oncological Targets. Oncogene 33, 5569–5581. 10.1038/onc.2013.578 24469031

[B48] León-Aparicio D. Salvador C. Aparicio-Trejo O. E. Briones-Herrera A. Pedraza-Chaverri J. Vaca L. (2019). Novel Potassium Channels in Kidney Mitochondria: The Hyperpolarization-Activated and Cyclic Nucleotide-Gated HCN Channels. Int. J. Mol. Sci. 20, 4995. 10.3390/ijms20204995 PMC6834191 31601020

[B49] Lukowski R. Cruz Santos M. Kuret A. Ruth P. (2021). 'cGMP and Mitochondrial K(+) Channels-Compartmentalized but Closely Connected in Cardioprotection. Br. J. Pharmacol. 179, 2344–2360. 10.1111/bph.15536 33991427

[B50] Martinez-Espinosa P. L. Yang C. Gonzalez-Perez V. Xia X.-M. Lingle C. J. (2014). Knockout of the BK β2 Subunit Abolishes Inactivation of BK Currents in Mouse Adrenal Chromaffin Cells and Results in Slow-Wave Burst Activity. J. Gen. Physiol. 144, 275–295. 10.1085/jgp.201411253 25267913 PMC4178941

[B51] Meera P. Wallner M. Toro L. (2000). A Neuronal β Subunit (KCNMB4) Makes the Large Conductance, Voltage- and Ca 2+ -activated K + Channel Resistant to Charybdotoxin and Iberiotoxin. Proc. Natl. Acad. Sci. U.S.A. 97, 5562–5567. 10.1073/pnas.100118597 10792058 PMC25868

[B52] Nagy D. Gönczi M. Dienes B. Szöőr Á. Fodor J. Nagy Z. (2014). Silencing the KCNK9 Potassium Channel (TASK-3) Gene Disturbs Mitochondrial Function, Causes Mitochondrial Depolarization, and Induces Apoptosis of Human Melanoma Cells. Arch. Dermatol Res. 306, 885–902. 10.1007/s00403-014-1511-5 25318378

[B53] O-Uchi J. Ryu S.-Y. Jhun B. S. Hurst S. Sheu S.-S. (2014). Mitochondrial Ion Channels/transporters as Sensors and Regulators of Cellular Redox Signaling. Antioxidants Redox Signal. 21, 987–1006. 10.1089/ars.2013.5681 PMC4116125 24180309

[B54] Olszewska A. Szewczyk A. (2013). Mitochondria as a Pharmacological Target: Magnum Overview. IUBMB Life 65, 273–281. 10.1002/iub.1147 23441041

[B55] Paggio A. Checchetto V. Campo A. Menabò R. Di Marco G. Di Lisa F. (2019). Identification of an ATP-Sensitive Potassium Channel in Mitochondria. Nature 572, 609–613. 10.1038/s41586-019-1498-3 31435016 PMC6726485

[B56] Pastore D. Soccio M. Laus M. N. Trono D. (2013). The Uniqueness of the Plant Mitochondrial Potassium Channel. BMB Rep. 46, 391–397. 10.5483/bmbrep.2013.46.8.075 23977986 PMC4133908

[B57] Paventi G. Soldovieri M. V. Servettini I. Barrese V. Miceli F. Sisalli M. J. (2022). Kv7.4 Channels Regulate Potassium Permeability in Neuronal Mitochondria. Biochem. Pharmacol. 197, 114931. 10.1016/j.bcp.2022.114931 35085542

[B58] Pereira O. Kowaltowski A. J. (2021). Mitochondrial K+ Transport: Modulation and Functional Consequences. Molecules 26, 2935. 10.3390/molecules26102935 34069217 PMC8156104

[B59] Peruzzo R. Mattarei A. Azzolini M. Becker-Flegler K. A. Romio M. Rigoni G. (2020). Insight into the Mechanism of Cytotoxicity of Membrane-Permeant Psoralenic Kv1.3 Channel Inhibitors by Chemical Dissection of a Novel Member of the Family. Redox Biol. 37, 101705. 10.1016/j.redox.2020.101705 33007503 PMC7527709

[B60] Prosdocimi E. Checchetto V. Leanza L. (2019). Targeting the Mitochondrial Potassium Channel Kv1.3 to Kill Cancer Cells: Drugs, Strategies, and New Perspectives. SLAS Discov. 24, 882–892. 10.1177/2472555219864894 31373829

[B61] Queliconi B. B. Wojtovich A. P. Nadtochiy S. M. Kowaltowski A. J. Brookes P. S. (2011). Redox Regulation of the Mitochondrial KATP Channel in Cardioprotection. Biochimica Biophys. Acta. Mol. Cell Res. 1813, 1309–1315. 10.1016/j.bbamcr.2010.11.005 PMC3109179 21094666

[B62] Rotko D. Bednarczyk P. Koprowski P. Kunz W. S. Szewczyk A. Kulawiak B. (2020a). Heme Is Required for Carbon Monoxide Activation of Mitochondrial BKCa Channel. Eur. J. Pharmacol. 881, 173191. 10.1016/j.ejphar.2020.173191 32422186

[B63] Rotko D. Kunz W. S. Szewczyk A. Kulawiak B. (2020b). Signaling Pathways Targeting Mitochondrial Potassium Channels. Int. J. Biochem. Cell Biol. 125, 105792. 10.1016/j.biocel.2020.105792 32574707

[B64] Severin F. Urbani A. Varanita T. Bachmann M. Azzolini M. Martini V. (2022). Pharmacological Modulation of Kv1.3 Potassium Channel Selectively Triggers Pathological B Lymphocyte Apoptosis *In Vivo* in a Genetic CLL Model. J. Exp. Clin. Cancer Res. 41, 64. 10.1186/s13046-022-02249-w 35172855 PMC8848658

[B65] Siemen D. Loupatatzis C. Borecky J. Gulbins E. Lang F. (1999). Ca2+-Activated K Channel of the BK-Type in the Inner Mitochondrial Membrane of a Human Glioma Cell Line. Biochem. Biophysical Res. Commun. 257, 549–554. 10.1006/bbrc.1999.0496 10198249

[B66] Singh H. Li M. Hall L. Chen S. Sukur S. Lu R. (2016). MaxiK Channel Interactome Reveals its Interaction with GABA Transporter 3 and Heat Shock Protein 60 in the Mammalian Brain. Neuroscience 317, 76–107. 10.1016/j.neuroscience.2015.12.058 26772433 PMC4737998

[B67] Singh H. Lu R. Bopassa J. C. Meredith A. L. Stefani E. Toro L. (2013). mitoBK Ca Is Encoded by the Kcnma1 Gene, and a Splicing Sequence Defines its Mitochondrial Location. Proc. Natl. Acad. Sci. U.S.A. 110, 10836–10841. 10.1073/pnas.1302028110 23754429 PMC3696804

[B68] Singh H. Stefani E. Toro L. (2012). Intracellular BKCa(iBKCa) Channels. J. Physiol. 590, 5937–5947. 10.1113/jphysiol.2011.215533 22930268 PMC3530108

[B69] Smith C. O. Nehrke K. Brookes P. S. (2017). The Slo(w) Path to Identifying the Mitochondrial Channels Responsible for Ischemic Protection. Biochem. J. 474, 2067–2094. 10.1042/bcj20160623 28600454 PMC5568769

[B70] Stowe D. F. Gadicherla A. K. Zhou Y. Aldakkak M. Cheng Q. Kwok W.-M. (2013). Protection against Cardiac Injury by Small Ca2+-Sensitive K+ Channels Identified in guinea Pig Cardiac Inner Mitochondrial Membrane. Biochimica Biophys. Acta. Biomembr. 1828, 427–442. 10.1016/j.bbamem.2012.08.031 PMC3534888 22982251

[B71] Stram A. R. Payne R. M. (2016). Post-translational Modifications in Mitochondria: Protein Signaling in the Powerhouse. Cell. Mol. Life Sci. 73, 4063–4073. 10.1007/s00018-016-2280-4 27233499 PMC5045789

[B72] Szabò I. Leanza L. (2017). The Roles of Mitochondrial Cation Channels Under Physiological Conditions and in Cancer. Handb. Exp. Pharmacol. 240, 47–69. 10.1007/164_2016_92 27995386

[B73] Szabó I. Bock J. Grassmé H. Soddemann M. Wilker B. Lang F. (2008). Mitochondrial Potassium Channel Kv1.3 Mediates Bax-Induced Apoptosis in Lymphocytes. Proc. Natl. Acad. Sci. U.S.A. 105, 14861–14866. 10.1073/pnas.0804236105 18818304 PMC2567458

[B74] Szabò I. Bock J. Jekle A. Soddemann M. Adams C. Lang F. (2005). A Novel Potassium Channel in Lymphocyte Mitochondria. J. Biol. Chem. 280, 12790–12798. 10.1074/jbc.m413548200 15632141

[B75] Szabò I. Leanza L. Gulbins E. Zoratti M. (2012). Physiology of Potassium Channels in the Inner Membrane of Mitochondria. Pflugers Arch. - Eur. J. Physiol. 463, 231–246. 10.1007/s00424-011-1058-7 22089812

[B76] Szabo I. Zoratti M. Biasutto L. (2021). Targeting Mitochondrial Ion Channels for Cancer Therapy. Redox Biol. 42, 101846. 10.1016/j.redox.2020.101846 33419703 PMC8113036

[B77] Szabo I. Zoratti M. (2014). Mitochondrial Channels: Ion Fluxes and More. Physiol. Rev. 94, 519–608. 10.1152/physrev.00021.2013 24692355

[B78] Szewczyk A. Bednarczyk P. (2018). Modulation of the Mitochondrial Potassium Channel Activity by Infrared Light. Biophysical J. 114, 43a. 10.1016/j.bpj.2017.11.288

[B79] Szteyn K. Singh H. (2020). BKCa Channels as Targets for Cardioprotection. Antioxidants (Basel) 9, 760. 10.3390/antiox9080760 PMC7463653 32824463

[B80] Testai L. Barrese V. Soldovieri M. V. Ambrosino P. Martelli A. Vinciguerra I. (2016). Expression and Function of Kv7.4 Channels in Rat Cardiac Mitochondria: Possible Targets for Cardioprotection. Cardiovasc Res. 110, 40–50. 10.1093/cvr/cvv281 26718475

[B81] Testai L. Rapposelli S. Martelli A. Breschi M. C. Calderone V. (2015). Mitochondrial Potassium Channels as Pharmacological Target for Cardioprotective Drugs. Med. Res. Rev. 35, 520–553. 10.1002/med.21332 25346462

[B82] Toczylowska-Maminska R. Olszewska A. Laskowski M. Bednarczyk P. Skowronek K. Szewczyk A. (2014). 'Potassium Channel in the Mitochondria of Human Keratinocytes. J. Invest Dermatol 134, 764–772. 10.1038/jid.2013.422 24126847

[B83] Trono D. Laus M. N. Soccio M. Alfarano M. Pastore D. (2015). Modulation of Potassium Channel Activity in the Balance of ROS and ATP Production by Durum Wheat Mitochondria-An Amazing Defense Tool Against Hyperosmotic Stress. Front. Plant Sci. 6, 1072. 10.3389/fpls.2015.01072 26648958 PMC4664611

[B84] von Charpuis C. Meckel T. Moroni A. Thiel G. (2015). The Sorting of a Small Potassium Channel in Mammalian Cells Can Be Shifted between Mitochondria and Plasma Membrane. Cell Calcium 58, 114–121. 10.1016/j.ceca.2014.09.009 25449299

[B85] Walewska A. Krajewska M. Stefanowska A. Buta A. Bilewicz R. Krysiński P. (2022b). Methods of Measuring Mitochondrial Potassium Channels: A Critical Assessment. Int. J. Mol. Sci. 23, 1210. 10.3390/ijms23031210 35163132 PMC8835872

[B86] Walewska A. Szewczyk A. Koprowski P. (2018b). Gas Signaling Molecules and Mitochondrial Potassium Channels. Int. J. Mol. Sci. 19, 3227. 10.3390/ijms19103227 PMC6214077 30340432

[B87] Walewska A. Kulawiak B. Szewczyk A. Koprowski P. (2018a). Mechanosensitivity of Mitochondrial Large-Conductance Calcium-Activated Potassium Channels. Biochimica Biophys. Acta. - Bioenergetics 1859, 797–805. 10.1016/j.bbabio.2018.05.006 29775559

[B88] Walewska A. Szewczyk A. Krajewska M. Koprowski P. (2022a). Targeting Mitochondrial Large-Conductance Calcium-Activated Potassium Channel by Hydrogen Sulfide via Heme-Binding Site. J. Pharmacol. Exp. Ther. 381, 137–150. 10.1124/jpet.121.001017 35184043

[B89] Wiedemann N. Pfanner N. (2017). Mitochondrial Machineries for Protein Import and Assembly. Annu. Rev. Biochem. 86, 685–714. 10.1146/annurev-biochem-060815-014352 28301740

[B90] Wojtovich A. P. Sherman T. A. Nadtochiy S. M. Urciuoli W. R. Brookes P. S. Nehrke K. (2011). SLO-2 Is Cytoprotective and Contributes to Mitochondrial Potassium Transport. PLoS One 6, e28287. 10.1371/journal.pone.0028287 22145034 PMC3228735

[B91] Wojtovich A. P. Smith C. O. Haynes C. M. Nehrke K. W. Brookes P. S. (2013). Physiological Consequences of Complex II Inhibition for Aging, Disease, and the mKATP Channel. Biochimica Biophys. Acta. Bioenergetics 1827, 598–611. 10.1016/j.bbabio.2012.12.007 PMC3880126 23291191

[B92] Wrzosek A. Augustynek B. Zochowska M. Szewczyk A. (2020). Mitochondrial Potassium Channels as Druggable Targets. Biomolecules 10, 1200. 10.3390/biom10081200 PMC7466137 32824877

[B93] Wrzosek A. Galecka S. Zochowska M. Olszewska A. Kulawiak B. (2022). Alternative Targets for Modulators of Mitochondrial Potassium Channels. Molecules 27, 299. 10.3390/molecules27010299 35011530 PMC8746388

[B94] Zemel M. Angelin A. Potluri P. Wallace D. C. Fieni F. (2022). The Principal Mitochondrial K+ Uniport Is Associated with Respiratory Complex I. bioRxiv. 10.1101/2022.01.06.475251

[B95] Zhao L. Feng Z. Yang X. Liu J. (2016). The Regulatory Roles of O-GlcNAcylation in Mitochondrial Homeostasis and Metabolic Syndrome. Free Radic. Res. 50, 1080–1088. 10.1080/10715762.2016.1239017 27646831 PMC5466075

